# Event-Related Potentials to Changes in Sound Intensity Demonstrate Alterations in Brain Function Related to Depression and Aging

**DOI:** 10.3389/fnhum.2020.00098

**Published:** 2020-03-27

**Authors:** Elisa M. Ruohonen, Saara Kattainen, Xueqiao Li, Anna-Elisa Taskila, Chaoxiong Ye, Piia Astikainen

**Affiliations:** ^1^Department of Psychology, University of Jyväskylä, Jyväskylä, Finland; ^2^Institute of Brain and Psychological Sciences, Sichuan Normal University, Chengdu, China

**Keywords:** aging, auditory-evoked potentials, depression, intensity dependence, sensory gating

## Abstract

Measures of the brain’s automatic electrophysiological responses to sounds represent a potential tool for identifying age- and depression-related neural markers. However, these markers have rarely been studied related to aging and depression within one study. Here, we investigated auditory event-related potentials (ERPs) in the brain that may show different alterations related to aging and depression. We used an oddball condition employing changes in sound intensity to investigate: (i) sound intensity dependence; (ii) sensory gating; and (iii) change detection, all within a single paradigm. The ERPs of younger (18–40 years) and older (62–80 years) depressed female participants and age-matched non-depressed participants were measured. Intensity dependence was examined as the difference between N1 responses to repeated high- and low-intensity sounds, sensory gating as N1 responses to rare and repeated sounds, and change detection as indexed by the mismatch negativity (MMN). We found that intensity dependence was greater in older participants than younger ones, indicating effects related to aging but not to depression. For sensory gating, we found depression- and age-related alterations as increased N1 responses. No group differences were found for MMN. Although a sensory gating deficit was expected in older adults, this study is the first to demonstrate age-related overexcitability in sound intensity dependency. The results indicate that automatic brain responses to sound intensity changes are suitable for studying age- and depression-related neural markers but may not be sensitive enough to differentiate the effects of aging and depression.

## Introduction

The brain’s automatic responses to sounds, that is, auditory event-related potentials (ERPs), can be used to investigate alterations in brain function related to aging and depression. Modulations in brain responses related to auditory change detection have been reported in association with old age and depression; it has been suggested that these modulations indicate cognitive decline (Näätänen et al., 2011). However, to the best of our knowledge, no previous study has attempted to simultaneously investigate age- and depression-related alterations in automatic auditory ERPs by using a single stimulus paradigm.

There is some indication that certain features of automatic auditory processing may be stronger markers of aging and others of depression. Processing of sound intensity could be expected to reflect depression-related alterations because of its association with serotonergic function (intensity dependency, Hegerl et al., 2001), which has been implicated in the pathophysiology of depression (Coppen, 1967; Meltzer, 1990; Hasler, 2010). On the other hand, inhibitory deficits reflected by a failure to suppress responses to repeated sounds, i.e., sensory gating, is a well-known phenomenon in aging (for review, see Friedman, 2011). One method for investigating brain responses affected by aging and depression is the oddball paradigm, which can be used to measure change detection. In this paradigm, a rare deviant sound is presented randomly among repeated standard sounds. A change detection response called mismatch negativity (MMN) is automatically elicited at frontocentral electrode sites approximately 150–250 ms after the onset of the deviant stimulus (Näätänen et al., 1978, 2007).

Several studies have reported attenuated MMN in older adults (Czigler et al., 1992; Schroeder et al., 1995; Gaeta et al., 1998; Alain and Woods, 1999; Cooper et al., 2006; Čeponienė et al., 2008; for a review, see Näätänen et al., 2011), but studies on depression have shown mixed results. Some depression studies have indicated augmented MMN for frequency changes (Kähkönen et al., 2007; He et al., 2010; Restuccia et al., 2016), while others have found attenuated MMN amplitude for duration changes (Naismith et al., 2012; Qiao et al., 2013; Chen et al., 2015), or attenuated MMN for both duration and frequency changes (Takei et al., 2009) in depressed participants compared to controls. Furthermore, other studies examining duration and frequency changes (Umbricht et al., 2003) or intensity changes (Ruohonen and Astikainen, 2017) found no depression-related effects on MMN. These discrepancies may be related to differences in the type of sound change (frequency, duration or intensity) applied in the experiments. Notably, studies that used frequency changes in high-intensity sounds (60 dB above the hearing threshold, or sounds presented at 80 dB) have found augmented MMN in depressed participants (Kähkönen et al., 2007; He et al., 2010; Restuccia et al., 2016).

Our previous study applied intensity changes in an oddball condition (60 vs. 80 dB) and observed enhanced N1 but no alterations in MMN in participants experiencing their first depressive episode compared to non-depressed control participants (Ruohonen and Astikainen, 2017). N1 is a negative deflection measured at frontocentral scalp locations around 100 ms after stimulus onset (Näätänen and Picton, 1987). This N1 enhancement may reflect a serotonergic deficit, which has been related to the pathophysiology of depression (Coppen, 1967; Maes and Meltzer, 1995). A steep increase in N1 in response to an increase in stimulus intensity has previously been associated with low serotonergic function and a shallow increase in brain response indicates higher serotonergic activity (Hegerl and Juckel, 1993; Hegerl et al., 2001). However, despite the association between intensity dependence and serotonergic function, some studies have failed to find differences in intensity dependence between depressed participants and controls (Linka et al., 2007; Park et al., 2010; Jaworska et al., 2012). Others have found these differences only in specific samples of depressed participants (Gopal et al., 2004; Fitzgerald et al., 2009; Lee et al., 2014). There is, however, growing evidence that a steeper slope of responses to increasing intensity (stronger intensity dependence) is associated with better treatment response to antidepressants (selective serotonin reuptake inhibitors, or SSRIs; Gallinat et al., 2000; Lee et al., 2005, 2015; Juckel et al., 2007; Jaworska et al., 2013). To the best of our knowledge, the intensity dependence of the auditory response in older adults has not been studied. Since old age is also associated with a decline in serotonergic function (Meltzer et al., 1998; Rodríguez et al., 2012), investigating potential N1 alterations related to aging and/or depression within the same sample is meaningful. Intensity dependence alterations may be expected in a sample including older depressed adults because of the similar decline in serotonergic function that occurs with aging and depression; this could have a cumulative effect on brain function.

N1 responses can also be used to investigate inhibition deficits related to aging. Aging has been commonly associated with deficits in the automatic filtering of irrelevant auditory information, or sensory gating (Friedman, 2011), a term that originated in schizophrenia studies (Freedman et al., 1987). According to the inhibition deficit theory, older adults may struggle to inhibit attention to irrelevant information, which can hinder task-related information processing (Hasher and Zacks, 1988; Lustig et al., 2008). Sensory gating is commonly measured with a paired-click task in which two identical sounds are presented with a short interstimulus interval; sensory gating is measured by the reduction in brain response to the second click (Friedman, 2011). Oddball conditions have also been used to study sensory gating. In such studies, a sensory gating deficit in older adults is indicated by larger N1 responses to repeated sounds in older compared to younger adults (Anderer et al., 1996; Amenedo and Díaz, 1998; Alain and Woods, 1999; Strömmer et al., 2017; for a review, see Friedman, 2011; for absent group difference, see Gaeta et al., 1998; Čeponienė et al., 2008). Although sensory gating deficit has also been associated with some neuropsychiatric disorders such as schizophrenia and bipolar disorder (Boutros et al., 1999, 2004; Lijffijt et al., 2009), the evidence on depression is scarce (see however, Baker et al., 1990; Wang et al., 2009). No previous studies have investigated sensory gating in older adults with depression.

This study will investigate whether brain responses measured within one stimulus paradigm can reveal markers related to aging and/or depression. Intensity changes will be presented in an oddball condition; which enables the investigation of sensory gating, intensity dependence and MMN within the same experiment. As mentioned above, sensory gating deficit has been demonstrated in aging (Anderer et al., 1996; Amenedo and Díaz, 1998; Alain and Woods, 1999; Strömmer et al., 2017; for a review, see Friedman, 2011), and intensity dependence may be a relevant marker for depression (Gopal et al., 2004; Fitzgerald et al., 2009; Lee et al., 2014). Furthermore, deficits in auditory change detection as indexed by MMN have been associated with both aging (Czigler et al., 1992; Schroeder et al., 1995; Gaeta et al., 1998; Alain and Woods, 1999; Cooper et al., 2006; Čeponienė et al., 2008) and depression (Kähkönen et al., 2007; He et al., 2010; Naismith et al., 2012; Qiao et al., 2013; Chen et al., 2015; Restuccia et al., 2016).

To investigate the effects related to aging and depression, we will compare the brain responses of older (over 61 years) and younger (18–40 years) participants with and without elevated symptoms of depression. First, we expect to observe age-related effects on sensory gating, specifically, we expect larger N1 responses in older participants than younger ones (for a review, see Friedman, 2011). Second, we hypothesize that the depression-related effects in intensity dependence will be found in older depressed adults, because both aging and depression can alter serotonergic function (Maes and Meltzer, 1995; Meltzer et al., 1998; Rodríguez et al., 2012). Perhaps this effect will not be observed in younger depressed participants since previous studies of younger participants have found alterations in intensity dependence only in some subgroups of depressed participants (Gopal et al., 2004; Fitzgerald et al., 2009; Lee et al., 2014). Alteration in change detection (MMN) is expected related to aging, but also possibly related to depression. Older adults are expected to have attenuated MMN amplitude compared to younger adults (Näätänen et al., 2011); it is less clear whether this effect is found related to depression since our previous study found no depression-related alteration in intensity MMN when examining young and middle-aged participants (Ruohonen and Astikainen, 2017). We will also examine the correlation between brain responses and cognitive test performance because previous studies have found an association between sensory gating and attentional functions (Erwin et al., 1998; Wan et al., 2008; Jones et al., 2016), between intensity dependence and behavioral inhibition (Kim et al., 2016), and between MMN amplitude and cognitive function (for a review, see Näätänen et al., 2011).

## Materials and Methods

### Participants

Female volunteers were recruited as study participants *via* newspaper advertisements, notice board advertisements and the University of Jyväskylä’s email lists. The participants were recruited as a part of research projects exploring the effects of interventions on aging-related cognitive changes and depression. Only females were recruited because our previous studies have shown that most of the volunteers for intervention studies are female and also because recruiting participants of both genders would increase sample heterogeneity. The experiment was conducted following the Declaration of Helsinki, and the ethical committee of the Central Finland Central Hospital approved the research protocol. Written informed consent was obtained from all of the participants before measurements were made.

Depressed and non-depressed participants in two age groups were recruited: younger adults aged 18–40 years and older adults over 61 years. Additional inclusion criteria for all participants were female gender, right-handedness, and normal hearing. Participants’ hearing thresholds were measured using a SA-51 audiometer (Mediroll Medico-Technical Limited); both ears were measured individually. Participants with a hearing threshold above 20 dB hearing level (HL) for 1,000 Hz sounds were excluded. The exclusion criteria for all the groups were self-reports of brain damage, current substance abuse, or neurological disorders (except migraine that was not recently active, learning disabilities or fibromyalgia) and ongoing psychological treatment (because the participants were recruited for a study examining effects of psychological interventions). The inclusion criterion for the depressed groups was current depressive symptoms (a score over 13 on a depression scale, which is the limit for mild depression; Beck et al., 1996). Depressive symptoms were measured using the BDI-II (Beck et al., 1996). The exclusion criteria for the depressed groups were a self-reported diagnosis of schizophrenia or bipolar disorder or a history of electroconvulsive therapy treatment. The exclusion criteria for the non-depressed groups were a self-reported current or previous diagnosis of depression, any other psychiatric diagnosis, current use of medication that can affect the central nervous system, and a BDI-II score over nine. This limit in BDI-II scores was chosen to ensure that the groups had clearly different numbers of depressive symptoms.

A total of 117 participants volunteered for the study: 23 younger adults (YOUNG), 22 younger adults with depression (YOUNG-D), 30 older adults (OLD), and 42 older adults with depression (OLD-D). In YOUNG, one participant was excluded because of a previous psychiatric diagnosis, and one participant dropped out of the study after recruitment. In YOUNG-D, three participants were excluded because of low depression scores and one because of handedness; two canceled their participation. In OLD, five participants were excluded because of hearing thresholds or use of a hearing device, two because of neurological disorders, one because of depression, two because of antidepressant use and two because of scheduling issues. In OLD-D, seven participants were excluded because of high hearing thresholds, seven because of low depression scores, one because of left-handedness, two because of ongoing psychological treatment (criterion for the intervention study), one because of active migraine and one because of scheduling issues and two canceled their participation.

After data collection, the data for two participants in OLD-D were excluded from further analysis because of excessive artifacts or a lack of visible obligatory responses (including N1) in the data. Also, the data for one participant from YOUNG and one from OLD were omitted because of technical problems during the electroencephalography (EEG) recording.

The final study included 20 participants in YOUNG, 16 in YOUNG-D, 17 in OLD, and 19 in OLD-D. Six participants in YOUNG-D reported a current or previous comorbid psychiatric diagnosis aside from depression: one reported an eating disorder, four reported anxiety disorders, and one reported a personality disorder. In OLD-D, one participant reported a previous diagnosis with a personality disorder. These participants were included in the sample because these comorbidities are common with depression. The demographics and clinical information for each group are provided in Table 1.

**Table 1:** Demographics and clinical variables for each group.

	YOUNG (*n* = 20)	YOUNG-D (*n* = 16)	OLD (*n* = 17)	OLD-D (*n* = 19)	Comparisons	T, (95% CI)	*p*
Age M ± SD (range)	27.5 ± 6.6 (19–39)	27.9 ± 6.9 (18–40)	67.5 ± 4.0 (63–80)	68.0 ± 4.3 (62–76)	YOUNG vs. YOUNG-D	*t*_(34)_ = 0.19, (−4.14, 5.02)	0.847
					OLD vs. OLD-D	*t*_(34)_ = 0.34, (−2.37, 3.31)	0.738
Education (low/medium/high)	0/6/14	0/8/8	3/7/7	2/12/4*	YOUNG vs. YOUNG-D	Na.	0.187
					OLD vs. OLD-D	Na.	0.340
					Younger vs. Older	Na.	0.008
					dep vs. non-dep	Na.	0.150
Diagnosis (within 1 year/over 1 year)	Na.	8/4	Na.	2/9	YOUNG-D vs. OLD-D	Na.	0.036
Depression severity (mild/moderate/severe)	Na.	0/7/1	Na.	1/6/1	YOUNG-D vs. OLD-D	Na.	1.000
Depression onset (childhood/adulthood)	Na.	8/4*	Na.	3/14*	YOUNG-D vs. OLD-D	Na.	0.008
Episodes (one/multiple)	Na.	6/10	Na.	3/15*	YOUNG-D vs. OLD-D	Na.	0.250
Medication (Non-med/med)	Na.	7/9	Na.	16/3	YOUNG-D vs. OLD-D	Na.	0.030
BDI-II M ± SD (range)	2.4 ± 1.9 (0–5)*	27.9 ± 7.4 (19–43)	3.5 ± 2.8 (0–9)	23.5 ± 5.7 (14–34)	YOUNG vs. OLD	*t*_(35)_ = 1.4, (−2.63, 0.49)	0.171
					YOUNG-D vs. OLD-D	*t*_(33)_ = 2.0, (−0.10, 8.91)	0.055
DASS-A M ± SD (range)	0.8 ± 0.8 (0–2)	10.5 ± 8.3 (1–38)	2.1 ± 1.2 (0–4)	6.2 ± 4.3 (1–17)*	YOUNG vs. OLD	*t*_(35)_ = 4.0, (−1.99, −0.65)	<0.001
					YOUNG-D vs. OLD-D	*t*_(32)_ = 1.9, (−0.29, 8.84)	0.065
SCL-90 som M ± SD (range)	2.6 ± 2.2 (0–8)	12.4 ± 9.7 (2–32)	4.2 ± 3.2 (0–10)	10.2 ± 7.6 (0–25)*	YOUNG vs. OLD	*t*_(35)_ = 2.0, (−3.47, 0.12)	0.066
					YOUNG-D vs. OLD-D	*t*_(32)_ = 0.8, (−3.72, 8.37)	0.439
BDI somatic (range)	Na.	8.5 ± 2.9 (4–13)	Na.	7.9 ± 2.4 (2–13)	YOUNG-D vs. OLD-D	*t*_(33)_ = 0.68, (−1.22, 2.43)	0.504
MMSE (range)	Na.	Na.	28.8 ± 1.4 (27–30)	29.1 ± 1.0 (25–30)*	OLD vs. OLD-D	*t*_(32)_ = 0.66, (−1.16, 0.59)	0.512

Abbreviations: DASS-A, Depression, anxiety and stress subscale (Lovibond and Lovibond, 1995); SCL-90 som, somatic subscale of the symptom checklist (Derogatis et al., 1973); MMSE, Mini-Mental State Examination (Folstein et al., 1975); BDI somatic, sum of scores on BDI-II items related to somatic symptoms (Buckley et al., 2001); diagnosis, number of participants with a self-reported diagnosis of a major depressive disorder within 1 year or over a year ago; severity, number of participants with a diagnosis of mild, moderate or severe depression (note that four participants in YOUNG-D and three in OLD-D could not specify their depression diagnoses); onset, number of participants who reported first onset of depressive symptoms in childhood (before the age of 18) or in adulthood; non-med/med, participants currently taking (med) and not taking (non-med) antidepressant medication; M, mean; SD, standard deviation; *data missing for participants (data about depression onset were missing for four participants in YOUNG-D and two participants in OLD-D; data about education, depressive episodes, DASS, SCL-90, and MMSE were missing for one participant in OLD-D). Note that the group comparisons with categorical variables are based on a two-tailed Fisher’s exact test; therefore, no “t-values” are reported for them. YOUNG, younger adult group; YOUNG-D, younger adult depression group; OLD, older adult group; OLD-D, older adult depression group; Younger, non-depressed and depressed younger adult groups; Older, non-depressed and depressed older adult groups; dep, depressed younger and older adults; non-dep, non-depressed younger and older adults; 95% CI, 95% confidence interval; *p*, p-value.

To examine differences in the groups’ hearing thresholds, a repeated measures of multivariate analysis of variance (MANOVA) was applied with a within-subjects factor ear (left vs. right) and with between-subjects factors age (younger vs. older) and depression (non-depressed vs. depressed). The MANOVA showed a significant main effect of age group, *F*_(1,68)_ = 29.1, *p* < 0.001, $\eta_{\text{p}}^{2}$ = 0.30, but no significant effect of depression group (*p* = 0.291), and no interaction effect between age group and depression group (*p* = 0.947) was found. The older groups had larger hearing thresholds (*M* = 7.6 dB, SD = 6.5; average calculated across the left and right ear) than the younger groups (*M* = 0.1 dB, SD = 4.9).

### Procedure

During the EEG measurement, the participant sat in a chair in a dimly lit, soundproof, electrically shielded room and was monitored through a camera positioned above a screen. The participant was instructed to watch a silent movie on the screen and ignore the sound stimuli played through a loudspeaker, which was located approximately one meter above the participant. The performance of various cognitive tests was measured on a different day. The results and analyses of the cognitive tests are provided in the Supplementary Material Section 1. The cognitive tests are described in Supplementary Table S1. A principal component analysis was conducted to form factors of the cognitive tests. The factor loadings for the cognitive tests and factor scores for younger and older group are reported in Supplementary Tables S2, S3, respectively.

### Stimulus Presentation

Sinusoidal sounds (1,000 Hz, 100 ms, 10 ms onset/offset ramps) were created with the Sound Forge program version 8.0 (Magix Software GmbH, Berlin, Germany), and presented with E-Prime 2.0 software (Psychology Software Tools Inc., Sharpsburg, MD, USA). Sound pressure levels (SPL) were measured with a sound level meter (type 2235, Brüel and Kjaer, Naerum, Denmark) with A-weighting.

The stimuli were presented in an oddball condition: a repeated standard sound was occasionally and randomly replaced by a deviant sound (presentation rate of 10%) of different intensity. The stimuli were presented in a pseudorandom order; at least two standard sounds were presented between the deviant sounds. The Stimulus Onset Asynchrony (SOA) was randomly set to either 500 ms, 550 ms or 600 ms. Longer SOAs would result in longer measurement time, which could be inconvenient for older adults. Much shorter SOAs were not possible, because there could be ongoing activity related to MMN and P3a components approximately at 150–250 ms (Näätänen et al., 1978, 2007) and 250–500 ms (Squires et al., 1975; Polich, 2007) latency after stimulus onset, respectively, that would be present at the baseline with a short SOA. Similar SOAs to those applied here has been used in previous studies of sensory gating (Strömmer et al., 2017), intensity dependence (Linka et al., 2007; Park et al., 2010; Lee et al., 2014) and MMN (He et al., 2010; Naismith et al., 2012; Chen et al., 2015; Ruohonen and Astikainen, 2017).

Two stimulus conditions were presented: increment and decrement conditions. In the increment condition, the standard stimulus was 60 dB (SPL) and the deviant stimulus was 80 dB (SPL). In the decrement condition, these intensities were reversed. Two blocks (each containing 450 standard and 50 deviant sounds) were present for both stimulus conditions. A program with one increment and one decrement block presented in random order was run twice, resulting in four possible combinations of presentation orders; these presentations varied randomly among the participants.

### Electroencephalography (EEG) Recording and Analysis

The EEG was measured using the Net Amps 200 (Electrical Geodesic Inc., Eugene, OR, USA) amplifier and a 128-channel sensor net (HydroCel Geodesic Sensor Net). The data were recorded with Net Station software (version 4.2.1). The sampling rate was 1,000 Hz, and online filters were set from 0.1 Hz to 400 Hz. The data were referenced online to a vertex electrode (Cz).

The data were analyzed with BrainVision Analyzer 2.1 (Brain Products GmbH, Munich, Germany). The data were filtered with a low cut off at 0.1 Hz and a high cut off at 30 Hz, a roll-off of 24 dB/octave, and a notch filter of 50 Hz. Eye movement artifacts were rejected through ocular correction *via* independent component analysis (ICA) as implemented in the BrainVision Analyzer. This procedure automatically detects ICA components. The representations for horizontal and vertical eye movements were manually selected from the ICA components based on a visual inspection. Channels with excessive noise were interpolated with a spherical spline model. The data were segmented into 600 ms segments (100 ms before and 500 ms after stimulus onset), and a period of 100 ms before stimulus onset was used as the baseline for the baseline correction. Segments with signal amplitudes outside −150 μV to 150 μV within a time period of 200 ms and segments with a difference of more than 50 μV between two consecutive time points were omitted from the analysis. Averages were calculated separately for three different stimulus types: responses to deviants, responses to standards immediately preceding the deviants (pre-deviant standard) and responses to standards immediately after deviants (post-deviant standard). The data were then re-referenced to an average of all channels. The average number of accepted trials calculated over all the stimulus types was 93.1 (SD = 7.7).

The channels and time windows were chosen based on previous literature (Näätänen and Picton, 1987; Näätänen, 1990; Gudlowski et al., 2009; Park et al., 2010; Ruohonen and Astikainen, 2017) and a visual inspection of the grand-averaged waveforms and topographies of the activity calculated across the groups. The same time windows and channels were found to fit both N1 sensory gating and intensity dependence responses. The N1 responses were calculated as the mean amplitude values of signals occurring 80–130 ms interval after stimulus onset over a frontocentral channel cluster of eight electrodes (the Fz electrode was the most frontal channel; see Supplementary Figure S1 and Supplementary Table S4). For MMN, mean amplitude values for deviant and pre-deviant standards were extracted from the frontal channel cluster (see Supplementary Figure S1 and Supplementary Table S4) 140 ms to 180 ms after stimulus onset.

### Statistical Analysis

The statistical analyses were conducted using IBM SPSS Statistics 24.0 (IBM Inc., Armonk, NY, USA). Separate analyses were conducted for N1 sensory gating, N1 intensity dependence, and MMN change detection.

For N1 sensory gating, responses to both standard and deviant sounds (see also Anderer et al., 1998; Tusch et al., 2016; Strömmer et al., 2017) for the conditions (increment and decrement) were included. For standard sounds, only responses to pre-deviant standards were included in the analysis because they had been repeated at least twice and therefore enabled an examination of the neural suppression related to stimulus repetition.

Intensity dependence was operationalized as the difference between N1 amplitudes in response to low- and high-intensity standard sounds. Only responses to post-deviant standard sounds were included in this analysis to reduce the effect of repetition on the responses. This is also more comparable to those of intensity dependence studies in which sounds are presented with equal probability and without repetition in consecutive stimuli. The differential response reflects the change in responses as a function of an increase in intensity, and therefore resembles a regression slope that is often used to study the intensity dependence of auditory responses (Hegerl et al., 1994). A similar analysis was performed by Gopal et al. (2004), who also calculated intensity dependence as the difference between responses to the sounds with the highest and lowest intensity.

The MMN response was calculated as a differential response by subtracting responses to the standard sounds from responses to the deviant sounds. Only responses to pre-deviant standard sounds were used in the analysis to ensure similar signal-to-noise ratios for standard and deviant responses. Separate differential waveforms were calculated for the increment (high-intensity deviant minus low-intensity standard) and decrement (low-intensity deviant minus high-intensity standard) conditions. In an additional analysis, MMN was calculated as the difference between the responses to a sound of the same intensity presented as a deviant and as a standard. The differential responses were calculated separately for the two oddball series (low-intensity sound as deviant and low-intensity sound as standard; high-intensity sound as deviant and high-intensity sound as standard). One advantage of this analysis is that it can control for sound intensity.

For sensory gating, a repeated measures of MANOVA was performed with between-subjects factors age group (younger vs. older adults) and depression group (depressed vs. non-depressed participants) and two within-subjects factors (stimulus type: standard vs. deviant, and condition: increment vs. decrement). For intensity dependence, an analysis of variance (ANOVA) was conducted to investigate the effects of age group and depression group on the N1 differential response. For MMN (differential response: deviant minus standard), a repeated measure of MANOVA with between-subject factors of age group and depression group and a within-subjects factor condition (increment vs. decrement) was performed. In the additional analysis for MMN, similar repeated measures of MANOVA was applied, but MMN was calculated as the difference between the responses to sounds of the same intensity; the within-subjects factor was intensity (high vs. low intensity). Because differential responses were applied in the MMN analyses, we also tested whether an MMN was elicited by comparing the responses to standards and deviants (averaged across the increment and decrement conditions) with a paired samples *t*-test.

To control for the effects of hearing thresholds and medication, separate analyses were conducted using the hearing threshold (the average threshold for the left and right ears) and medication status (current use of antidepressant medication vs. no medication) as covariates. A multivariate analysis of covariance (MANCOVA) was conducted for sensory gating and MMN and analysis of covariance (ANCOVA) for intensity dependence.

Whenever significant interaction effects with stimulus type and either of the group factors (age or depression) were observed, they were followed by two-tailed independent samples *t*-tests comparing the groups for each stimulus type [corrected using false discovery rate (FDR); Benjamini and Hochberg, 1995].

*P*-values under 0.05 were considered significant. The partial eta squared was reported as the effect size for the MANOVA and ANOVA and Cohen’s *d* with a pooled standard deviation (Cohen, 1988) for pairwise comparisons.

We tested whether the number of accepted trials differed across stimulus types by conducting two separate repeated measures of MANOVAs corresponding to the statistical models of the brain response analyses. The same model was used for sensory gating and MMN since they had the same stimulus types and pre-processing, and thus identical trials in the brain response analyses. The accepted trials for responses to deviant stimuli and for pre-deviant standard stimuli, which corresponded to the sensory gating and MMN analyses, were compared using repeated measures of MANOVAs with two between-subjects factors (age and depression) and two within-subject factors (stimulus type and condition). There were no significant main or interaction effects between the trial numbers (all *p*s > 0.050). For intensity dependence, the trial numbers for post-deviant standard stimuli were applied since intensity dependence was calculated from these responses. The trial numbers for post-deviant standard stimuli, which corresponded to the intensity dependence analysis, were compared using a repeated-measures of MANOVA between the groups (age and depression) with a within-subject factor condition (increment vs. decrement). There were no significant main or interaction effects between the trial numbers (*p*s > 0.300).

## Results

### Intensity Dependence of the N1 Responses

**Figure 1:**
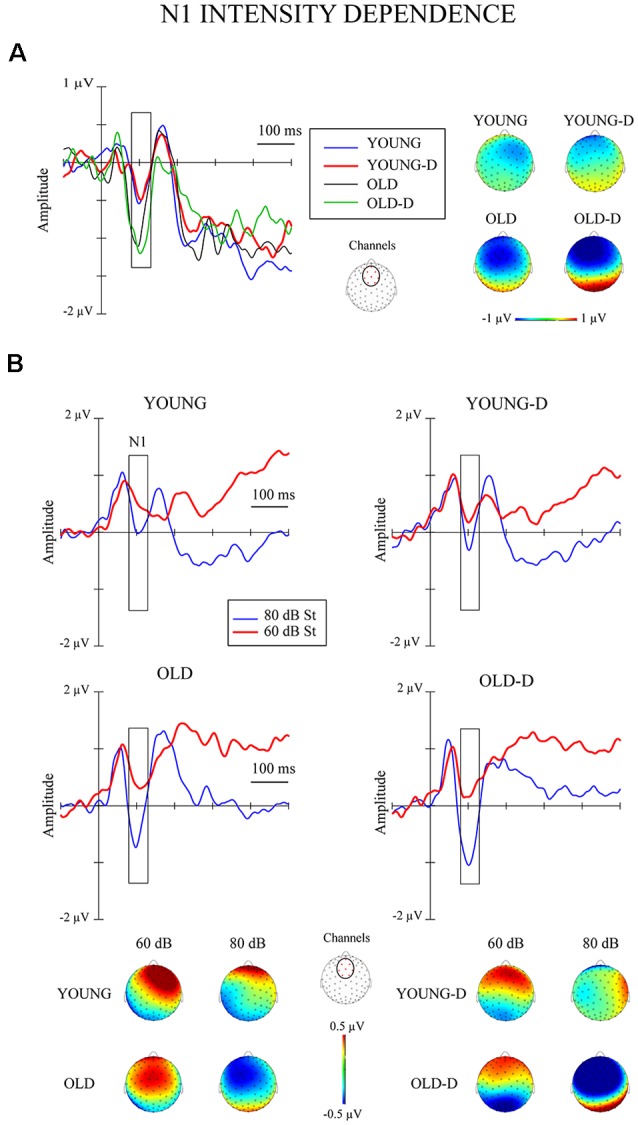
Intensity dependence responses. **(A)** N1 differential responses. Waveforms for the N1 differential responses (high-intensity standard minus low-intensity standard sounds) and topographical maps of the responses for each group. **(B)** N1 responses to standards. The grand-averaged waveforms over the frontocentral channel cluster for responses to low- and high-intensity standard sounds and topographical maps of the responses. The rectangles overlaid with the waveforms show the time window applied in the analyses (mean amplitude values between 80 ms and 130 ms after stimulus onset). The topographies represent the average amplitude in the same time window. The channel cluster applied in the analysis is marked in the figure between the topographies. St = response to standard sound, YOUNG = younger adult group, YOUNG-D = younger adult depression group, OLD = older adult group, OLD-D = older adult depression group.

The grand-averaged waveforms and topographical maps for the analysis of N1 intensity dependence are shown in Figure 1. The significant ANOVA/ANCOVA effects are shown in Table 2.

**Table 2:** The significant effects for intensity dependence and sensory gating investigated with ANOVA/MANOVA and ANCOVA/MANCOVA with hearing threshold and medication as covariates.

Response	Model	Effect	*F*-value	*df*	*p*-value	$\eta_{\text{p}}^{2}$
Intensity dependence	ANOVA	age	9.7	1,68	0.003	0.12
	ANCOVA hear thres*	age	14.4	1,67	<0.001	0.18
		Hearing threshold	4.3	1,67	0.043	0.06
	ANCOVA medication	age	6.8	1,67	0.012	0.10
Sensory gating	MANOVA	age	11.3	1,68	0.001	0.14
		Age × stimulus type × condition	7.2	1,68	0.009	0.10
	MANCOVA hear thres*	age	8.0	1,67	0.006	0.11
		Age × stimulus type × condition	7.0	1,67	0.011	0.10
	MANCOVA medication	age	7.0	1,67	0.011	0.10
		Depression	5.4	1,67	0.024	0.07
		Age × stimulus type × condition	7.0	1,67	0.010	0.10
	MANOVA excl med^+^	age	6.5	1,56	0.013	0.11
		Depression	4.3	1,56	0.043	0.07
		Age × condition	6.3	1,56	0.015	0.10
		Age × stimulus type × condition	4.8	1,56	0.032	0.08

Notes. *hear thres, hearing threshold as covariate; ^+^excl med, MANOVA without medicated depressed participants; df, degrees of freedom; $\eta_{\text{p}}^{2}$, partial eta squared.

**Figure 2:**
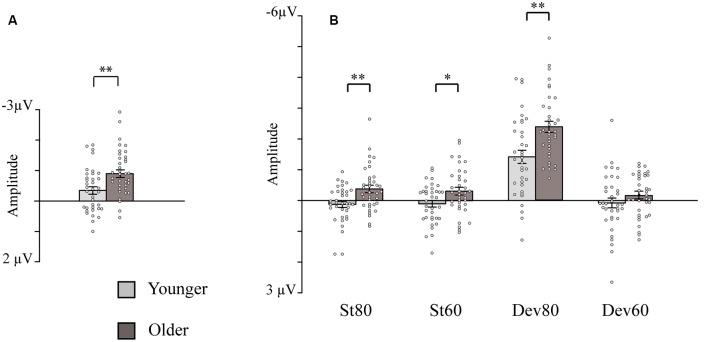
Bar charts showing the amplitudes of the intensity dependence responses and N1 responses (sensory gating). **(A)** Intensity dependence: Mean amplitude values for the N1 differential responses (high-intensity standard minus low-intensity standard response). **(B)** Sensory gating: Mean amplitude values for the N1 responses to the four stimulus types. Mean amplitudes are presented separately for the younger and older groups (depression group collapsed). Error bars represent standard error. *p < 0.05, **p < 0.01. St80, standard sound of 80 dB; St60, standard sound of 60 dB; Dev80, deviant sound of 80 dB; Dev60, deviant sound of 60 dB; Younger, YOUNG and YOUNG-D collapsed; Older, OLD and OLD-D collapsed.

The ANOVA examining the effect of age group and depression group on N1 intensity dependence showed a significant main effect of age group (Table 2), but no significant effect of depression group (*p* = 0.614), or age group × depression group interaction effect (*p* = 0.407), were found. Larger responses were found in the older group (*M* = −0.9 μV, SD = 0.8) than in the younger group (*M* = −0.3 μV, SD = 0.7; see Figure 2A).

Separate ANCOVAs were conducted to investigate the effect of hearing threshold and current medication status on responses by adding these factors as covariates to the models comparing the groups. The age group effect remained significant when controlling for the hearing threshold and when controlling for medication status (Table 2), but there were still no main or interaction effects of the depression group (*p*s > 0.400). However, there was a significant main effect of the hearing threshold (Table 2).

Pearson correlations were conducted between cognitive factors (memory, processing speed, verbal fluency, working memory, and inhibition; see Supplementary Material Section 1.1) and intensity dependence across all participants whose data were available for cognitive tests (*n* = 65). There were no significant correlations (*p*s > 0.170).

### N1 Sensory Gating

**Figure 3:**
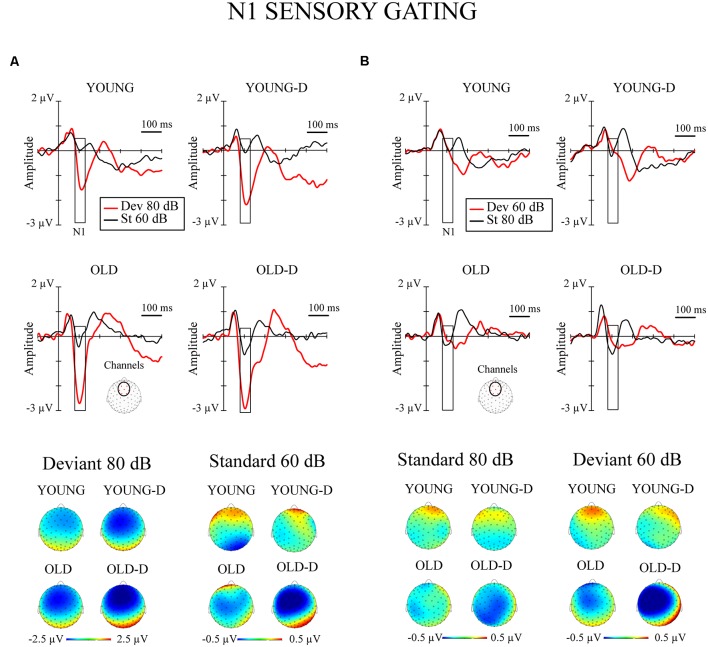
N1 sensory gating. Grand-averaged waveforms of the N1 responses (sensory gating) from the frontocentral electrode sites (channels) and the corresponding topographical maps. **(A)** Increment condition. **(B)** Decrement condition. The rectangles overlaid with the waveforms show the time window applied in the analyses (mean amplitude values between 80 -130 ms after stimulus onset), and the topographies represent the average amplitude over the same time window. The channel cluster applied in the analysis is marked to the figure below the waveforms. YOUNG, younger adult group; YOUNG-D, younger adult depression group; OLD, older adult group; OLD-D, older adult depression group. Dev 80 dB, 80 dB deviant sound; St 60 dB, 60 dB standard sound; Dev 60 dB, 60 dB deviant sound; St 80 dB, 80 dB standard sound.

The grand-averaged waveforms and topographical maps for N1 responses to deviant and standard sounds are shown in Figure 3. The significant MANOVA/MANCOVA effects are shown in Table 2.

The repeated measures of MANOVA examining the effect of age group and depression group on N1 sensory gating revealed a significant stimulus type × condition × age group interaction and the main effect of age group (Table 2). No significant main or interaction effects of depression group were found (*p*s > 0.130). The main effect of age group was found as larger responses in older group (*M* = −0.8 μV, SD = 0.6) compared to younger group (*M* = −0.3 μV, SD = 0.7).

The effects of hearing threshold and medication status were examined by adding these factors as covariates to separate MANCOVA’s comparing the groups. The stimulus type × condition × age group interaction effect and the main effect of age group remained significant when controlling for hearing threshold and medication status (Table 2). No significant main effect of depression group or interaction effects with depression group were found when controlling for the hearing threshold (*p*s > 0.100). However, the main effect of the depression group was found when controlling for medication (Table 2). The depressed had larger N1 responses to all stimuli (M_adj_ = −0.8 μV, SD = 0.7) than the non-depressed (M_adj_^1^ = −0.4 μV, SD = 0.7). To further investigate the effect of medication, the original mixed-model MANOVA was performed, excluding medicated participants. The stimulus × condition × age group and the main effect of age group remained significant (Table 2), but there was also a main effect of depression group. Non-medicated depressed participants had larger N1 amplitudes (*M* = −0.9 μV, SD = 0.6) than non-depressed participants (*M* = −0.4 μV, SD = 0.7).

The *post hoc* tests for the significant stimulus type × condition × age group effect were conducted with independent sample *t*-tests comparing the responses of the older and younger groups (collapsed over depression groups) to the four different stimulus types. An FDR correction was used to correct for multiple comparisons (four tests). Older adults had larger N1 amplitudes to high-intensity standards, low-intensity standards, and high-intensity deviants than younger adults; no group difference was observed for low-intensity deviants (Table 3, Figure 2B).

**Table 3:** The *post hoc* tests investigating age group × stimulus type × condition interaction in sensory gating. The mean (M) amplitude values (μV), standard deviations (SD) and independent samples *t*-tests comparing the mean amplitudes between the younger and the older group.

Stimulus type	Group	M (SD)	*t*-value	*p*-value	*df*	Cohen’s *D*	95% CI
st 80 dB	Younger	0.1 (0.6)	3.2	0.004	70	0.8	0.19, 0.81
	Older	−0.4 (0.7)					
st 60 dB	Younger	0.1 (0.6)	2.5	0.018	70	0.6	0.09, 0.74
	Older	−0.3 (0.8)					
dev 80 dB	Younger	−1.4 (1.3)	3.4	0.004	70	0.8	0.40, 1.52
	Older	−2.4 (1.1)					
dev 60 dB	Younger	0.1 (1.0)	1.2	0.221	70	0.4	−0.15, 0.63
	Older	−0.2 (0.7)					

*Notes. Df, degrees of freedom; 95% CI, 95% confidence interval; St 80 dB, 80 dB standard sound; St 60 dB, 60 dB standard sound; dev 80 dB, 80 dB deviant sound; dev 60 dB, 60 dB deviant sound*.

Pearson correlations were conducted between cognitive factors (memory, processing speed, verbal fluency, working memory, and inhibition; see Supplementary Material Section 1.1) and sensory gating (average calculated over stimulus types) across all participants whose data were available for cognitive tests (*n* = 65). There were no significant correlations (*p*s > 0.400; FDR corrected).

### MMN Responses

**Figure 4:**
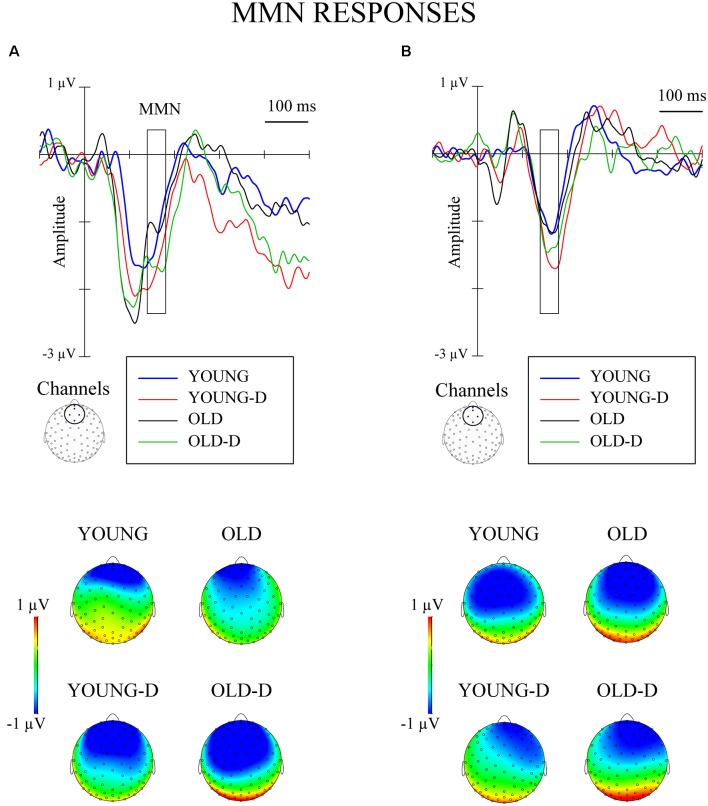
Grand-averaged waveforms for mismatch negativity (MMN; deviant–standard from the same oddball series) from a frontocentral channel cluster and topographical maps. **(A)** Increment condition. **(B)** Decrement condition. The rectangles overlaid with the waveforms and the topographies show the time window applied in the analyses (mean amplitude values between 140 ms and 180 ms after stimulus onset). YOUNG, younger adult group, YOUNG-D, younger adult depression group; OLD, older adult group; OLD-D, older adult depression group.

The waveforms and topographies for the MMN calculated as the difference between the responses to the standard and the deviant stimuli obtained from the same oddball series are shown in Figure 4.

Repeated measures of MANOVA for the MMN differential responses (stimuli obtained from the same oddball series) showed no significant main or interaction effects (*p*s > 0.250). No effects of depression or age group were found when controlling for hearing threshold or medication (*p*s > 0.200). However, the MMN was elicited: larger responses were found for the deviant (*M* = −0.7 μV, SD = 1.3) than the standard stimuli (*M* = 0.6 μV, SD = 0.7) across groups, *t*_(71)_ = 10.7, 95% CI (1.07, 1.56), *p* < 0.001.

The MMN was also analyzed as the difference between responses to stimuli of the same intensity presented as a standard and as a deviant. Repeated measures of MANOVA revealed no significant main or interaction effects (*p*s > 0.160).

Pearson correlations were conducted between cognitive factors (memory, processing speed, verbal fluency, working memory, and inhibition; see Supplementary Material Section 1.1) and MMN (average of both conditions) across all participants whose data were available for cognitive tests (*n* = 65). There were no significant correlations (*p*s > 0.240).

## Discussion

This study investigated age- and depression-related alterations in the ERPs to sound intensity changes. Sensory gating, intensity dependence, and change detection were compared for groups of older and younger depressed and non-depressed participants. We found age-related alterations in sensory gating and intensity dependence. Depression-related effects were found for sensory gating when controlling for medication status or when only non-medicated depressed participants were included in the analysis.

Contrary to our expectations, no depression-related effects were observed for intensity dependence, but instead, an effect of age was found. This was indicated by the larger difference in older adults’ N1 amplitudes to repeated high- and low-intensity sounds compared to younger adults (when the responses were averaged across the depressed and non-depressed participants). To the best of our knowledge, this is the first study investigating intensity dependence in older adults. However, the association between age and intensity dependence has been studied in younger samples (mean age ranged from 39 to 50 years in previous studies; Linka et al., 2007, 2009; Park et al., 2010; Min et al., 2012). One study found an association between age and weaker intensity dependence (Min et al., 2012); others have found no association between age and N1 responses (Linka et al., 2007, 2009; Park et al., 2010). However, since previous studies did not include older participants, it is difficult to compare their findings to ours. Since stronger intensity dependence may reflect a deficit of serotonergic function (Hegerl et al., 2001), the present finding of greater intensity dependence in older adults might reflect a decrease in serotonergic function in this group. This finding aligns with previous studies that have demonstrated an age-related decrease in serotonergic activity (for reviews, see Meltzer et al., 1998; Rodríguez et al., 2012). However, conclusions should be made cautiously, because direct support for the relationship between monoamine function and intensity dependence has as yet only been found in animal studies (Juckel et al., 1997, 1999). Furthermore, the results of human studies have been inconclusive and sometimes have even contradicted the intensity dependence hypothesis (Dierks et al., 1999; Debener et al., 2002; Kähkönen et al., 2002; Massey et al., 2004).

The null finding in this study for depression related to intensity dependence aligns with some previous studies that also found no differences between depressed participants and controls (Linka et al., 2007; Park et al., 2010; Jaworska et al., 2012). It is also possible that the heterogeneity of the illness profile within the depression group masked the depression-related effects in the present study. There is evidence that intensity dependence is weaker in melancholic than in non-melancholic depressed participants (Fitzgerald et al., 2009). Another study found that intensity dependence was stronger in depressed participants with atypical depression compared to participants with non-atypical depression (Lee et al., 2014). If intensity dependence differs based on the depression subtype, the heterogeneity in the depression samples may explain the discrepancies among studies. To clarify this point, more research is needed comparing participants with different depression profiles.

As expected, an age-related effect was found for sensory gating. Older adults had larger N1 amplitudes to both high- and low-intensity standard sounds compared to younger adults, which aligns with previous studies (Anderer et al., 1996; Amenedo and Díaz, 1998; Alain and Woods, 1999; Strömmer et al., 2017) and suggests a deficit of inhibitory control (Friedman, 2011). In addition to enlarged N1 responses to standard sounds, the older adults displayed larger N1 responses to rare high-intensity sounds. This is a novel finding related to aging. Only a few previous studies have investigated N1 sensory gating as a response to deviant sounds (Anderer et al., 1998; Strömmer et al., 2017). These studies found an age-related augmentation of repeated sounds, but not of rare sounds. The experimental design by Anderer et al. (1998) can be considered the most similar to our study since it employed intensity changes whereas the other sensory gating studies employed frequency changes. That study investigated responses to repeated high-intensity sounds and rarely presented low-intensity sounds. Age-related augmentation was specific to repeated high-intensity sounds and not found for rare low-intensity sounds (Anderer et al., 1998). In contrast, we found that older adults had increased N1 responses to both repeated sounds and rare high-intensity sounds. Similarly, one previous study found an age-related increase in N1 amplitude for both repeated sounds and novel sounds (Tusch et al., 2016). The authors suggest that the increase in these responses could reflect an adaptive response to compensate for reduced processing speed in older adults. Our finding of augmented N1 in older adults could reflect a similar compensatory effect. Another explanation is that the larger N1 responses relate to the increased distractibility associated with aging, since it has been suggested that N1 reflects pre-attentive attention switching (Näätänen, 1990).

In addition to age-related effects, an alteration related to depression was found in N1 responses, but only when the effect of medication was controlled for or when only non-medicated participants’ data were included. In these analyses, depressed participants had larger N1 responses compared to non-depressed participants across both stimulus types. Since larger responses occurred with both repeated and rare stimuli, this result could reflect general cortical overexcitability in depression rather than a deficit in sensory gating. The reason why this effect was only observed when investigating non-medicated depressed participants could be that antidepressant medication might correct this overexcitability. In our previous study, which used the same stimulus paradigm with a younger sample (18–64 years, *M* = 42 years; Ruohonen and Astikainen, 2017), we also found a relationship between depression and increased N1 responses, but only in the processing of rare deviant sounds. It is not clear why these depression-related effects were observed in the current study for both standard and deviant stimuli. However, one possible explanation for the discrepancy between the current results and the previous finding is differences in the study samples. The present study included older participants (18–80 years; *M* = 48 years), whereas the previous study included young and middle-aged participants (Ruohonen and Astikainen, 2017). Also, the depressed sample in the present study included mostly participants with recurrent depression; in the previous study, only the participants with first-episode depression but not the participants with recurrent depression differed from controls (Ruohonen and Astikainen, 2017).

We conclude that it may not be feasible to use sensory gating to differentiate the effects of aging and depression since we found increased responses in both older adults and depressed adults, and no cumulative effect of aging and depression were observed (i.e., larger N1 amplitude in the group of older depressed adults than in older non-depressed adults).

The finding of larger MMN responses to deviant and standard sounds across groups indicates that the MMN was elicited, but no group differences in MMN response were observed. This contrasts with previous studies that have found MMN attenuation in older adults (Czigler et al., 1992; Schroeder et al., 1995; Gaeta et al., 1998; Alain and Woods, 1999; Cooper et al., 2006; Čeponienė et al., 2008). However, some studies have not found age-related MMN attenuation to sound duration or frequency changes when applying relatively short interstimulus intervals (Pekkonen et al., 1993, 1996; Strömmer et al., 2017), similar to ones applied in our study. Our results cannot be directly compared to those of previous studies because we applied an intensity change detection condition, whereas previous studies have employed sound frequency or duration changes. Perhaps the intensity changes are not the most prominent way to demonstrate age-related alterations in change detection—at least when applying short interstimulus intervals.

We did not find any depression-related alterations in MMN responses, either. This aligns with our previous study, which applied the same stimulus conditions and found no differences in MMN between first-episode depressed, recurrent depressed and non-depressed young and middle-aged adults (Ruohonen and Astikainen, 2017). Previous studies have found inconsistent results for the modulation of MMN in depression; some have found attenuated MMN in depressed participants (Takei et al., 2009; Naismith et al., 2012; Qiao et al., 2013; Chen et al., 2015); some have found augmented MMN (Kähkönen et al., 2007; He et al., 2010; Restuccia et al., 2016); and some have found no difference in MMN between depressed and controls (Umbricht et al., 2003). The discrepancies among the studies could be related to differences in the applied stimuli or the type of deviance. Studies that associated augmented MMN with depression applied frequency changes and relatively high stimulus intensities (Kähkönen et al., 2007; He et al., 2010; Restuccia et al., 2016); studies that associated attenuated MMN with depression mostly applied duration changes (Naismith et al., 2012; Qiao et al., 2013; Chen et al., 2015). Possibly, the intensity change condition (used in the present study) is not the best tool for detecting depression-related effects on the MMN time window.

The discrepancies among MMN studies could also be related to sample characteristics. Some studies included only non-medicated depressed participants, while others included both medicated and non-medicated participants. Although, we found that medication did not affect MMN responses when it was added as a covariate, MMN modulation might be observed in a sample of only non-medicated depressed participants. Some previous studies have included participants with specific types of depression or additional deficits, such as treatment-resistant depression (He et al., 2010), melancholic depression (Chen et al., 2015), first-episode depression without a treatment history (Qiao et al., 2013), or older depressed adults with mild cognitive impairment (MCI; Naismith et al., 2012). Our sample included participants with a range of depression histories (including differences in symptom duration and recurrence) and possibly different depression subtypes.

This study has some limitations. The sample size was relatively small, so, possibly some effects were too small to be detected. Only female participants were recruited for this study, and therefore, the findings only apply to females. On one hand, the inclusion of only female participants could be considered a strength as it reduces the heterogeneity of the sample. On the other hand, some of the discrepancies between our findings and those of previous studies could be related to our inclusion of only females in this study. The female gender has been associated with weaker intensity dependence only in one previous study (Min et al., 2012). Some studies have found stronger intensity dependence in females (Hensch et al., 2008; Oliva et al., 2011; Jaworska et al., 2012) and most of the other studies have found no relationship between gender and intensity dependence (Juckel et al., 2007; Linka et al., 2007; Park et al., 2010). Therefore, our inclusion of only female participants may not explain the null findings related to intensity dependence and depression. Previous studies investigating gender effects on MMN have been inconclusive (see e.g., Barrett and Fulfs, 1998; Kasai et al., 2002; Ikezawa et al., 2008; Matsubayashi et al., 2008), so it is difficult to determine whether the female sample could explain the lack of an effect of age or depression on MMN.

Out of 35 depressed participants, 23 participants reported a diagnosis of a major depressive disorder. Clearer effects may have been observed with a sample consisting only of participants who are clinically diagnosed with depression. Another limitation is that the study included participants currently taking antidepressant medication (12 of 35). Future studies should confirm the findings by examining participants with no recent or current antidepressant use.

We used a novel stimulus condition to study intensity dependence, which is usually measured in experimental conditions in which several different stimulus intensities are presented in random order. We used an oddball condition in which two different stimulus intensities, one designated as rare (deviant) and one as repeated (standard), were presented. This protocol was used to enable our study to investigate sensory gating, change detection and intensity dependence using a single experiment. However, to make the study more comparable to previous studies of intensity dependence, we analyzed the responses to the standard sounds presented immediately after deviant sounds to minimize the effect of repetition on the responses. Future studies should directly compare intensity dependence analyzed using this oddball condition and using traditional intensity dependence stimulus conditions.

The results of this study indicate that automatic auditory ERP responses to intensity changes can be used to study changes in brain function related to aging and depression. However, the method did not differentiate the effects of depression from those of age. Intensity changes were used because this condition made it possible to simultaneously investigate intensity dependence, change detection, and sensory gating. It is possible that applying frequency or duration MMN conditions, which have previously shown age- (for a review, see Näätänen et al., 2011) and depression-related effects (Kähkönen et al., 2007; He et al., 2010; Naismith et al., 2012; Chen et al., 2015; Restuccia et al., 2016), could enable differentiation of the groups. Frequency or duration changes presented in oddball condition would still enable a simultaneous investigation of sensory gating and change detection, but not of intensity dependence. Another option would be to use frequency change detection conditions employing high-intensity stimuli (90 dB). Since previous studies have found augmented frequency MMN in depressed participants when using high-intensity stimuli (Kähkönen et al., 2007; He et al., 2010; Restuccia et al., 2016) and attenuated MMN has been found in general in older adults (Näätänen et al., 2011), this experimental condition might show different effects on MMN for aging and depression (decreased amplitude for older adults and increased amplitude for depressed adults).

One clinically relevant question for future studies is whether an intensity oddball condition could be used to distinguish the effects related to MCI from those of late-onset depression, since both conditions are commonly associated with somewhat similar cognitive decline and also depressive symptoms (Steffens, 2008; Pellegrino et al., 2013; Leyhe et al., 2017). The automatic auditory responses applied here have been associated with cognitive function (Erwin et al., 1998; Wan et al., 2008; Näätänen et al., 2011; Jones et al., 2016), so it may be expected that these responses would be more affected in groups with greater cognitive deficits (i.e., a larger difference might be expected between depressed older adults and participants with MCI than between depressed older adults and healthy older adults). There is some evidence that participants with MCI have sensory gating deficits (for a review, see Friedman, 2011) and attenuated or absent MMN compared to healthy controls (Mowszowski et al., 2012; Lindín et al., 2013; Ruzzoli et al., 2016). However, previous studies have not attempted to distinguish patients with MCI from those with late-onset depression.

In sum, we found evidence of age- and depression-related effects in auditory intensity processing by using a method that allowed a simultaneous investigation of sensory gating, intensity dependence, and change detection. However, none of the responses directly distinguished the age and depression groups. In both older adults and depressed adults, augmented N1 responses were found, indicating similar alterations in intensity processing in both groups. These alterations could be related to sensory gating deficits, or they might reflect a more general overexcitability in the processing of sounds since the effect was not specific for repeated sounds. In the depression group, this excitability was only observed in non-medicated participants, so the results highlight the importance of studying non-medicated groups when searching for biomarkers for depression. Unexpectedly, intensity dependence did not reveal depression-related but instead age-related effects. The greater intensity dependence found in older adults may reflect the serotonergic deficits that have previously been associated with aging (Meltzer et al., 1998; Rodríguez et al., 2012). The heterogeneity of symptom profiles and medication within the depression group could explain why no depression-related effects were observed. The stimulus paradigm should be further developed to optimize its ability to differentiate age and depression, and its ability to distinguish patients with MCI from those with depression could be tested.

## Data Availability Statement

The raw data supporting the conclusions of this article will be made available by the authors, without undue reservation, to any qualified researcher.

## Ethics Statement

The studies involving human participants were reviewed and approved by Central Finland Central Hospital, Jyväskylä. The patients/participants provided their written informed consent to participate in this study.

## Author Contributions

PA and ER contributed to the conception, design of the study and drafted the manuscript. ER, SK, XL, A-ET, and CY collected the data. ER analyzed the data. SK and A-ET contributed to the data analysis. All the authors contributed to the preparation of the manuscript and approved the submitted version of the manuscript.

## Conflict of Interest

The authors declare that the research was conducted in the absence of any commercial or financial relationships that could be construed as a potential conflict of interest.

## Footnotes

^1^M_adj_ = Mean value adjusted for medication status.
