# Supplementary material for: Event-Related Potentials to Changes in Sound Intensity Demonstrate Alterations in Brain Function Related to Depression and Aging
Source: Front Hum Neurosci. 2020 Mar 27;14:98. doi: 10.3389/fnhum.2020.00098 (PMC7119431; doi:10.3389/fnhum.2020.00098)
Supplement: Supplementary file 1 [file Data_Sheet_1.docx]

Supplementary Material

# Factor analysis for the cognitive tests

In this section, the formation of the factors based on the cognitive tests and the group comparisons of the factor scores are reported. A principal component analysis was conducted to form factors of the cognitive tests. Group differences in cognitive performance were examined by comparing the cognitive factors between the groups. It was hypothesized that the depressed groups would differ from the non-depressed groups and the older age groups would differ from the younger groups in memory, executive functions and attention, because dysfunction in these cognitive functions have been previously associated with depression (for reviews, see Castaneda et al., 2008; Elderkin-Thompson et al., 2011) and aging (for a review, see Harada et al., 2013).

## Cognitive tests

The participants attended a separate session for cognitive tests measuring memory, executive functions and attention. The tests were administered by students majoring in psychology, who had received training and supervision for the testing from a psychologist. The data for the cognitive tests were available from 18 YOUNG, 13 YOUNG-D, 18 OLD-D and 16 OLD participants. Altogether, 27 tests were administered to all the participants (Table 1). In addition, a Mini-mental state examination (MMSE) was employed for the older participants to ensure that no participants with scores indicating mild cognitive impairment (scores below 24) were included in the sample (the MMSE scores for the groups are reported in the main text Table 1).

A principal component analysis was performed to form factors of the cognitive test scores and reduce variables for the group comparisons. Missing data for the participants on an individual test was replaced by a mean value calculated based on all the participants’ data for that test. In OLD-D there were missing data for one participant in Trail Making-B test and for one participant in delayed logical memory and in the OLD for one participant in Stroop 3. The finger tapping test and a hand grip strength test were not included in the factor model because they measure motor performance that is conceptually distinct from the other cognitive measures. In addition, the Stroop 1 and Rey–Osterrieth complex figure copying tests **(**Rey) were excluded because they only served as practice tests for the following Stroop 2 and 3 tests and Rey memory recalls, respectively. For the initial exploratory factor analysis, 23 tests were added as variables to investigate factor solutions and the fit of each test for the solution. The exploratory analysis suggested six factors that had eigenvalue higher than 1.0 (oblimin with Kaiser normalization). A five-factor solution was attempted because, with the six-factor solution, one factor consisted mostly of one test only. Two variables (errors in the Stroop 2 test and errors in Stroop 3 test) were left out from the final five-factor model because they loaded to factors which they did not conceptually fit. The final solution included scores from 21 cognitive tests (see Table 2) that had communalities greater than 0.478 and explained 71.2% of the variance. The factors were named memory, processing speed, verbal fluency, working memory and inhibition. A repeated measures of MANOVA was conducted with between-subject factors age (younger vs. older) and depression (depressed vs. non-depressed) and a within-subject factor (cognitive factors which included five factors).

## Results of the group comparisons

A repeated measures of MANOVA comparing the effects of age group and depression group on the cognitive factors, revealed a significant age group x cognitive factor interaction effect, *F*(4, 58) = 14.9, *p* < .001, η^2^_p_ = 0.51 and a main effect of age group, F(1, 61) = 6.4, *p* = .014, η^2^_p_ = 0.10. The follow-up tests for the age group x cognitive factor interaction were conducted by comparing age groups (younger vs. older; collapsed over depression group) for each factor with the independent sample t-tests. The p-values were corrected with false discovery rate correction (Benjamini & Hochberg, 1995). The comparisons showed worse performance for the older group compared to the younger group in memory, t(63) = 3.3, *p* = .002, 95% CI [0.31, 1.23], in processing speed t(63) = -7.4, *p* < .001, 95% CI [-1.7, -1.00] and in inhibition, t(63) = -2.9, *p* = .008, 95% CI [-1.15, -0.21] (see Table 3). However, no significant differences were found for the fluency or working memory, *ps* = .080.

## Discussion

In agreement with previous studies that have found aging-related alterations in cognitive function (Harada et al., 2013), we found worse performance in older adults compared to younger adults for cognitive measures of memory, processing speed and inhibition. Against our expectation, we did not find depression-related effects in cognitive performance. However, the results related to the cognitive measures should be interpreted cautiously because the sample size was smaller than for the ERPs.

| **Supplementary Table 1.** Description of the cognitive tests. |
| --- |
| **The digit span task** (Wechsler Adult Intelligence Scale III [WAIS-III]): The test includes two subtests that measure short-term memory and working memory. In **the digit span forward**, a participant is asked to repeat a sequence of numbers that are read aloud to the participant. In **the digit span backward** the participant has to repeat the numbers in a reversed order. The tests are scored based on the number of correctly recalled sequences (Wechsler, 1997a). |
| **The digit symbol coding task** (WAIS-IV): In the task, a participant receives an A4 paper including numbers from 1 to 9 organized in several rows. Below the numbers are empty boxes and the participant is asked to draw a correct symbol, that matches the number, to each box. On top of the page, a key is provided that presents the correct symbol corresponding to each number. The test is scored based on the number of correctly filled symbols within two minutes. The task measures eye–hand coordination and processing speed (Wechsler, 2008). |
| **The finger tapping task:** A participant presses a button on a tally counter device, and the number of button presses produced within 10 seconds time is calculated. The tests are administered for the left and right hands. The test measures psychomotor speed and motor control (Ruff and Parker, 1993). |
| **Handgrip strength tests:** The test measures the maximum isometric strength of hand muscles. A participant holds a hydraulic dynamometer and is asked to squeeze the handle as hard as possible for approximately five seconds. The test is repeated three times for the left and right hands, and an average of these tests is calculated. Low grip strength has been associated with disability and mortality in old age (Bohannon, 2008). |
| **The letter–number sequencing** (Wechsler Memory Scale III): In the test, sequences of numbers and letters are read aloud to a participant. The participant is asked to repeat the series by first repeating the numbers from smallest to largest followed by the letters in alphabetical order (Wechsler, 1997b). |
| **The logical memory task** (Wechsler Memory Scale-Revised): The test includes two short stories that are read aloud to a participant. The participant is asked to repeat each story immediately after it is read and again approximately one hour after the story has been read. The tests measure immediate and delayed declarative memory (Wechsler, 1987). |
| **Minimental state examination (MMSE):** The test is a questionnaire that is used to measure cognitive impairment and screen for dementia. It measure attention, recall, calculation and orientation. Scores below 24 indicate cognitive impairment (Folstein et al., 1975). |
| **Rey auditory verbal learning test (RAVLT):** A list of 15 nouns is read to a participant, and the participant is asked to repeat the words. The process is repeated five times, each time reading the list aloud and then recording the correctly recalled words. The fifth recall represents total learning (RAVLT learning total). Then a list of 15 new words is read, and the participant is asked to repeat the words. The new list serves as a distraction. After this, the participant is asked to recall the first list again (RAVLT immediate recall). The test measures the distractibility of immediate memory recall. The participant is asked to repeat the list again approximately one hour later (delayed recall). (Schmidt, 1996). |
| **Rey–Osterrieth complex figure test (Rey’s figure):** The participant is shown a figure that includes several random shapes and asked to reproduce the figure by drawing it as accurately as possible. The participant is asked to draw the figure from memory immediately (immediate recall) and again approximately one hour after it was first presented (delayed recall). The participant is not notified beforehand of the memory recall tasks. The tasks measure visual memory (Shin, et al. 2006). |
| **Stroop color-word test:** The Stroop tests consist of three tests that measure executive functions. The Stroop 1 test includes color words and a participant is asked to read the words aloud as fast as possible. The Stroop 2 test includes groupings of letter “x” printed with blue, yellow and red ink on a sheet of paper. The participant is asked to name the color in which the letters are printed. In the Stroop 3 test, a list of color names is printed with incongruent ink color. The participant is asked to name the ink color and restrain from reading aloud the written word. The participant is asked to perform each task as fast and accurately as possible. The reading time and errors are recorded. The tasks measure attention, naming speed and inhibition (Alvarez and Emory, 2006). |
| **Symbol search (WAIS-IV):** In the task, a participant receives a notebook consisting of A4 pages. In each page, there are symbols organized in rows. In each row, two symbols are presented in the left column and a series of symbols on the right column. The participant is asked to cross over any symbol on the right (row) that corresponds to a symbol in the left. If none of the symbols match, the participant needs to cross a box tagged “no.” A two-minute time period is given, and the number of correctly identified symbols are recorded. The task measures processing speed (Wechsler, 2008). |
| **Trail making tests (TMT):** In the **TMT-A,** a participant receives an A4 paper that has numbers (1 to 25) printed in random locations. The participant is asked to connect the numbers by drawing straight lines in order from the smallest number to the largest number. In the **TMT-B**, the sheet includes letters from A to L and numbers from 1 to 13 in random locations. The task is to connect the items by alternating between numbers and letters and advancing in ascending and alphabetical order. Both tasks are to be performed as fast and accurately as possible. The time taken and errors are recorded. The tasks measure processing speed, executive functions and attention. The TMT-B is more demanding and requires divided attention, whereas the TMT-A measures more sustained attention and psychomotor speed **(**Bowie and Harvey, 2006). |
| **Verbal fluency:** A participant is asked to name as many words from a certain category as possible within one minute. The test is scored based on the number of correctly produced words. Three tasks are administered: producing words starting with the letter K and the letter S and naming animal species. The task measures phonemic fluency (letters K and S), semantic fluency (naming of animals) and executive functions (Lezak et al., 2012). |

**Supplementary Table 2**. Factor loadings for the principal components of the cognitive test scores

|  | Memory | | Processing speed | Verbal fluency | Working memory | Inhibition |
| --- | --- | --- | --- | --- | --- | --- |
| RAVLT immediate recall | **.849** | .087 | | .115 | .010 | -.096 |
| RAVLT delayed recall | **.792** | .022 | | .105 | -.058 | .111 |
| RAVLT learning total | **.782** | .074 | | .148 | -.058 | -.071 |
| REY figure delayed recall | **.602** | -.536 | | .046 | .055 | .038 |
| REY figure immediate recall | **.581** | -.514 | | .068 | .091 | .042 |
| Logical memory delayed | **.506** | -.242 | | -.027 | -.475 | -.003 |
| Logical memory immediate | **.500** | -.214 | | -.064 | -.429 | -.077 |
| Symbol search | .160 | **-.723** | | .139 | -.077 | .041 |
| Digit symbol | .209 | **-.704** | | .072 | -.084 | -.089 |
| TMT-A | .069 | **.679** | | .183 | .225 | .141 |
| Stroop 3 | .183 | **.584** | | -.337 | .099 | .329 |
| Fluency-S | .201 | .101 | | **.817** | -.091 | .064 |
| Fluency-K | .154 | .038 | | **.719** | -.159 | .035 |
| Fluency-animal | .287 | -.107 | | **.580** | -.001 | -.239 |
| Stroop 2 | .326 | .520 | | **-.550** | -.021 | .017 |
| Digit-letter | -.016 | -.249 | | -.019 | **-.801** | .042 |
| Digit span backward | .031 | .086 | | .018 | **-.775** | .012 |
| Digit span forward | -.134 | .092 | | .353 | **-.683** | -.119 |
| TMT-B errors | .079 | .209 | | -.063 | -.123 | **.859** |
| TMT-A errors | -.148 | -.243 | | .050 | .118 | **.718** |
| TMT-B | -.148 | .532 | | .129 | -.002 | **.535** |

Note that for each test, the largest factor loading is marked in bold to indicate the tests that had the greatest contribution for each component.

| **Supplementary Table 3.** The mean values and standard deviations for the cognitive factor scores with significant group differences. | | | |
| --- | --- | --- | --- |
| Group | Memory | Processing speed | Inhibition |
| Younger (n = 31) | 0.4 (0.7) | -0.7 (0.7) | -0.4 (0.3) |
| Older (n = 34) | -0.4 (1.1) | 0.6 (0.8) | 0.3 (1.3) |

Note that for processing speed the smaller the factor score the better the performance.

# Electrodes applied in the analyses


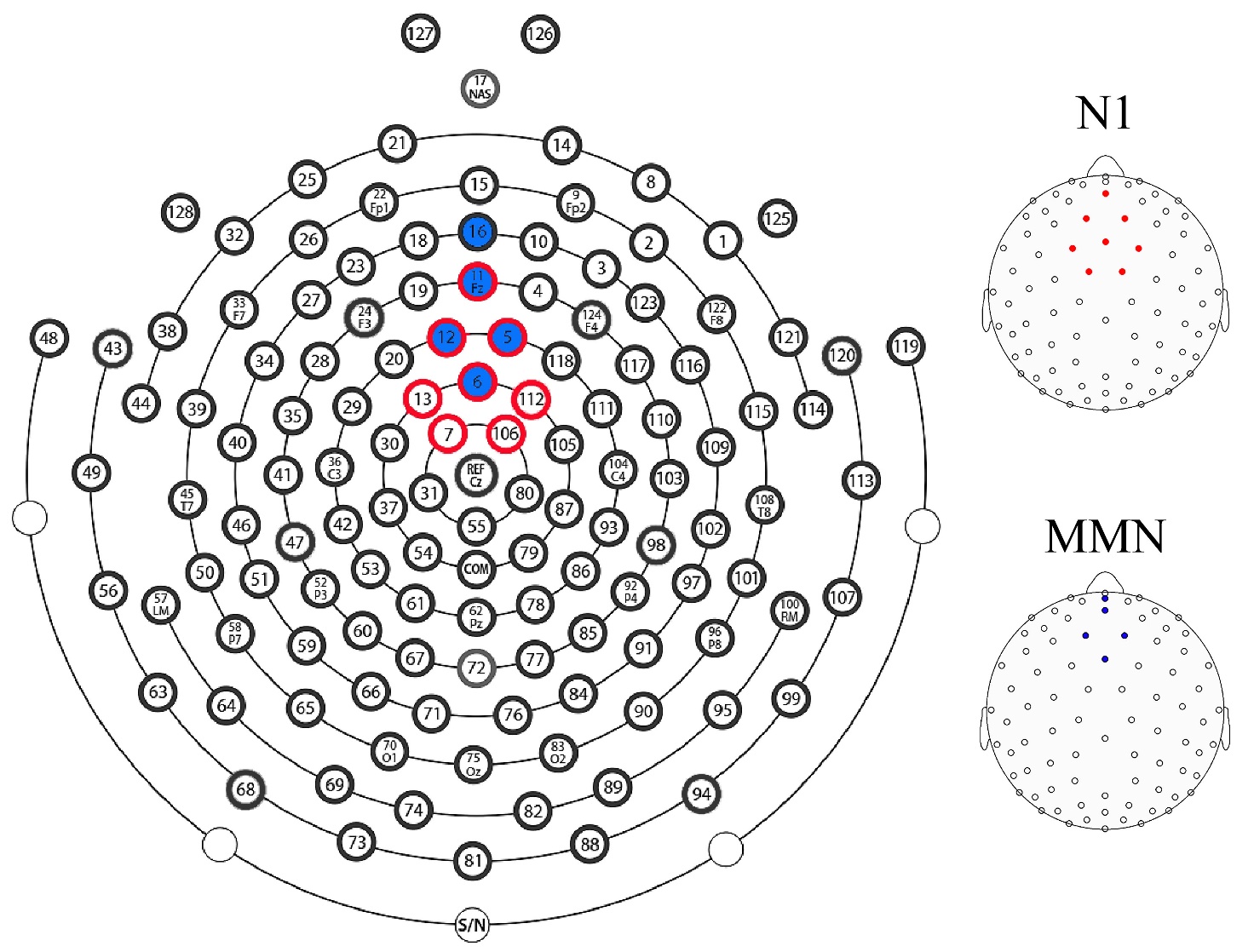


**Supplementary Figure 1.** HydroCel Geodesic Sensor Net 128-channel electrode cap (Electrical Geodesics Inc., OR, USA) and the topographic maps indicating the electrode clusters for N1 and MMN. In the sensor net (left), the channels for N1 are marked with red circles and the ones for the MMN, with blue filling. In the topographic maps (right), with red (N1) and blue (MMN) fillings.

**Supplementary Table 4.** The electrodes for N1 and MMN

| Component | Electrode cluster |
| --- | --- |
| N1 | 5, 6, 7, 11, 12, 13, 106, 112 |
| MMN | 5, 6, 11, 12, 16 |

# References

Alvarez, J. A., and Emory, E. (2006). Executive function and the frontal lobes: A meta-analytic review. *Neuropsychol. Rev*. 16, 17–42. https://doi.org/10.1007/s11065-006-9002-x

Benjamini, Y., and Hochberg, Y. (1995). Controlling the false discovery rate: a practical and powerful approach to multiple testing. *Source J. R. Stat. Soc. Ser. B 57*, 289–300. https://doi.org/10.2307/2346101

Bohannon, R.W. (2008). Hand-grip dynamometry predicts future outcomes in aging adults. J. *Geriatr. Phys. Ther.* 31, 3–10.

Bowie, C. R., and Harvey, P. D. (2006). Administration and interpretation of the Trail Making Test. *Nat. Protoc.* 1, 2277–2281. https://doi.org/10.1038/nprot.2006.390

Castaneda, A. E., Tuulio-Henriksson, A., Marttunen, M., Suvisaari, J., and Lönnqvist, J. (2008). A review on cognitive impairments in depressive and anxiety disorders with a focus on young adults. *J. Affect. Disord.* 106, 1–27. https://doi.org/10.1016/j.jad.2007.06.006

Elderkin-Thompson, V., Moody, T., Knowlton, B., Hellemann, G., and Kumar, A. (2011). Explicit and Implicit Memory in Late-Life Depression. *Am. J. Geriatr. Psychiatry* 19, 364–373. https://doi.org/10.1097/JGP.0b013e3181e89a5b

Folstein, M.F., Folstein, S.E., and McHugh, P.R. (1975). "Mini-mental state". A practical method for grading the cognitive state of patients for the clinician. *J. Psychiatr. Res.* 12, 189–98.

Harada, C. N., Natelson Love, M. C., and Triebel, K. L. (2013). Normal cognitive aging. *Clin. Geriatr. Med.* 29, 737–52. https://doi.org/10.1016/j.cger.2013.07.002

Lezak M., Howieson D., Bigler E., and Tranel, D. (2012). Neuropsychological Assessment. Oxford University Press, New York.

Ruff, R. M., and Parker, S. B. (1993). Gender- and age-Specific changes in motor speed and eye-hand coordination in adults: Normative values for the finger tapping and grooved pegboard tests. *Percept. Mot. Skills* 76, 1219–1230. https://doi.org/10.2466/pms.1993.76.3c.1219

Schmidt, M. (1996). Rey Auditory and Verbal Learning Test. A handbook. Western Psychological Association, Los Angeles.

Shin, M.-S., Park, S.-Y., Park, S.-R., Seol, S.-H., and Kwon, J. S. (2006). Clinical and empirical applications of the Rey–Osterrieth Complex Figure Test. *Nat. Protoc.* 1, 892–899. https://doi.org/10.1038/nprot.2006.115

Wechsler, D. (1987). Manual for the Wechsler Memory Scale-Revised, The Psychological Corporation, San Antonio, TX.

Wechsler, D. (1997a). WAIS‐III administration and scoring manual. Psychological Corporation, San Antonio, TX.

Wechsler, D. (1997b). Wechsler Memory Scale- Third Edition. Psychological Corporation, San Antonio, TX.

Wechsler, D. (2008). Wechsler adult intelligence scale–Fourth Edition (WAIS–IV). San Antonio, TX.

**
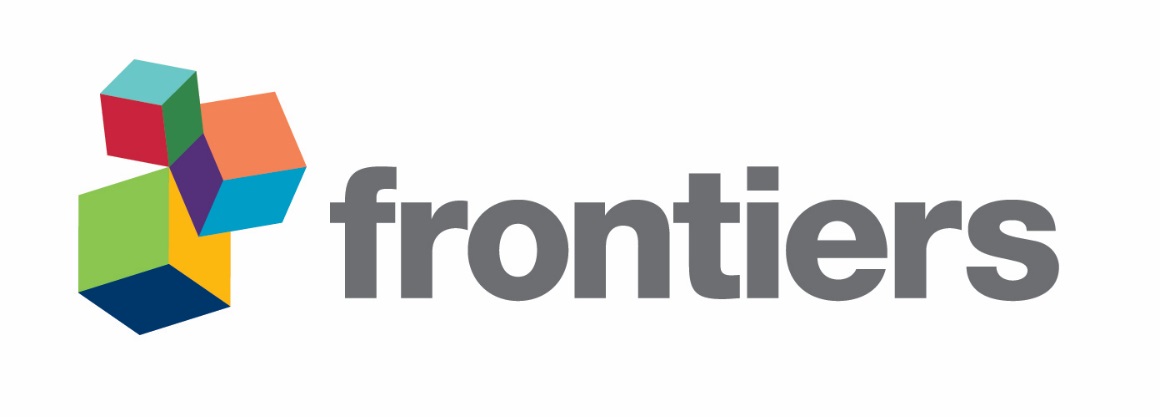
**
